# A Potentially Inexpensive Diagnostic Method for A2 Pulley Ruptures

**DOI:** 10.7759/cureus.5751

**Published:** 2019-09-25

**Authors:** Fenil Bhatt, Arij Batul, Francisco Schwartz-Fernandes

**Affiliations:** 1 Miscellaneous, University of South Florida Morsani College of Medicine, Tampa, USA; 2 Orthopaedics, University of South Florida Morsani College of Medicine, Tampa, USA

**Keywords:** flexor, tendon trauma, tendon, a2 pulley, hand surgery, flexor tendon

## Abstract

Injuries to the flexor pulley system of the hand, while uncommon, can be problematic and debilitating for patients. Current standards of diagnosis for A2 pulley disruptions often include costly imaging studies and inaccurate clinical testing. We present the case of a patient with an isolated complete A2 pulley avulsion that was diagnosed by employing a simple technique using a silicone wedding ring. A silicone wedding band was applied over the region of a suspected A2 pulley rupture, resulting in the immediate restoration of full range of motion as well as complete resolution of the injury after three months, without any need for surgical intervention. The usage of this ring confirmed an A2 pulley avulsion that was visualized on magnetic resonance imaging (MRI). The proposed “Wedding Band Test” is potentially an accurate and inexpensive diagnostic tool for clinical evaluation of A2 pulley ruptures.

## Introduction

The flexor pulley system is a crucial structure that provides for the full flexion of the fingers at the distal interphalangeal (DIP) and proximal interphalangeal (PIP) joints. Injuries to this system are more common in activities that place a great deal of stress on these joints. Of this system, the A2 pulley is considered to be the most crucial due to its significant load-bearing capabilities [1].

The increasing prevalence of activities such as rock climbing has led to an increase in injuries of the A2 pulley, likely due to the unique biomechanics of the "crimping" grip used in climbing [2]. One study reported a 58% incidence of flexor pulley injury as a result of rock climbing in a sample size of 623 patients, and of those patients, 52% involved the middle finger and 50% involved the A2 pulley [3].

Rupture or injury to the A2 and A4 pulley can result in a loss of torque at the DIP and PIP joints, along with a phenomenon known as “bowstringing”, in which the affected finger fails to flex fully and is associated with pain or discomfort. This is associated with a significant decrease in work and excursion efficiency [4]. These ruptures can occur when a sudden extension of the digit is preceded by a large external load onto the flexed digit, such as in a rock climbing or martial arts accidents [2].

## Case presentation

**Figure 1:**
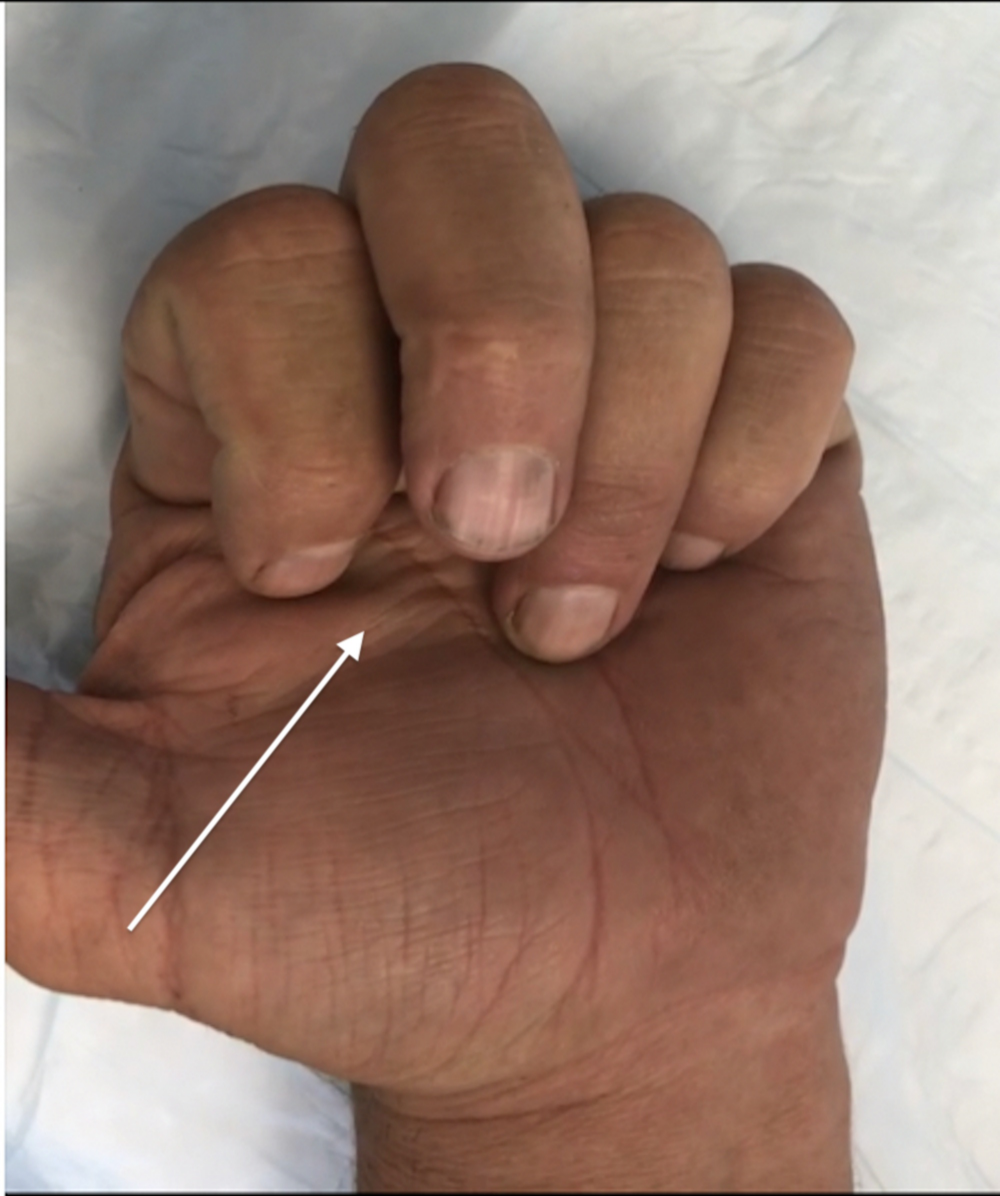
Partial flexion before silicone ring placement

**Figure 2:**
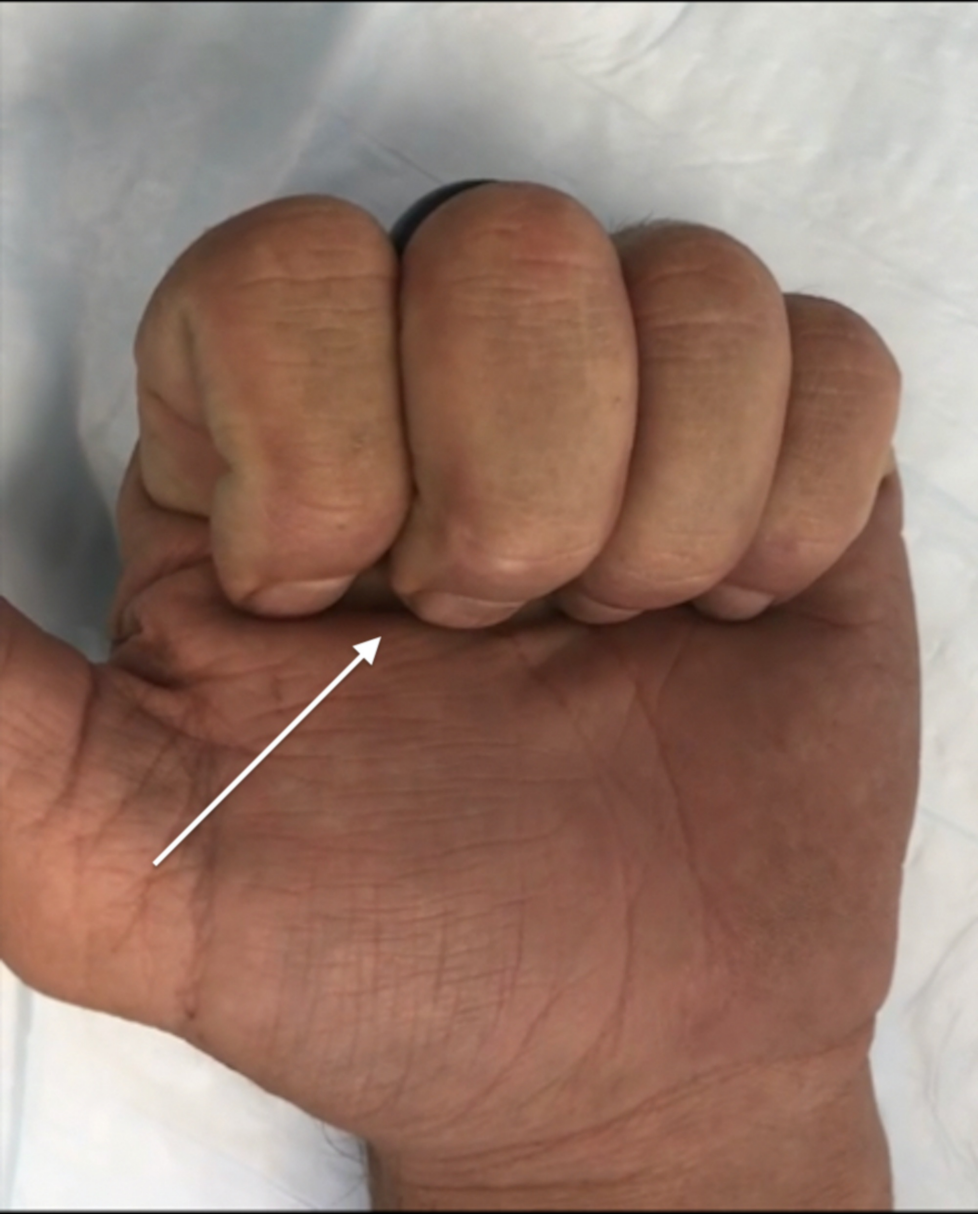
Full flexion with silicone ring placement

**Figure 3:**
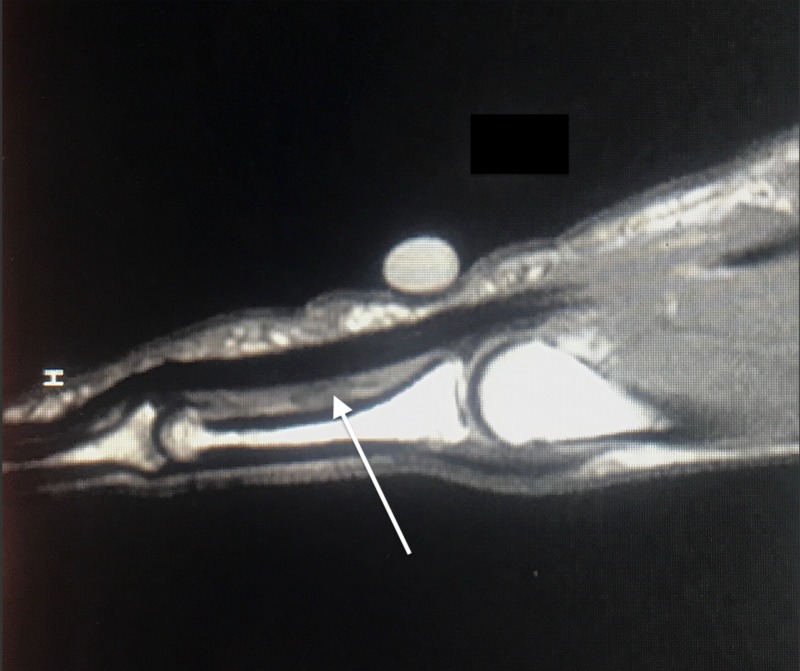
Separation of tendon from phalanx at the region of the A2 pulley

**Figure 4:**
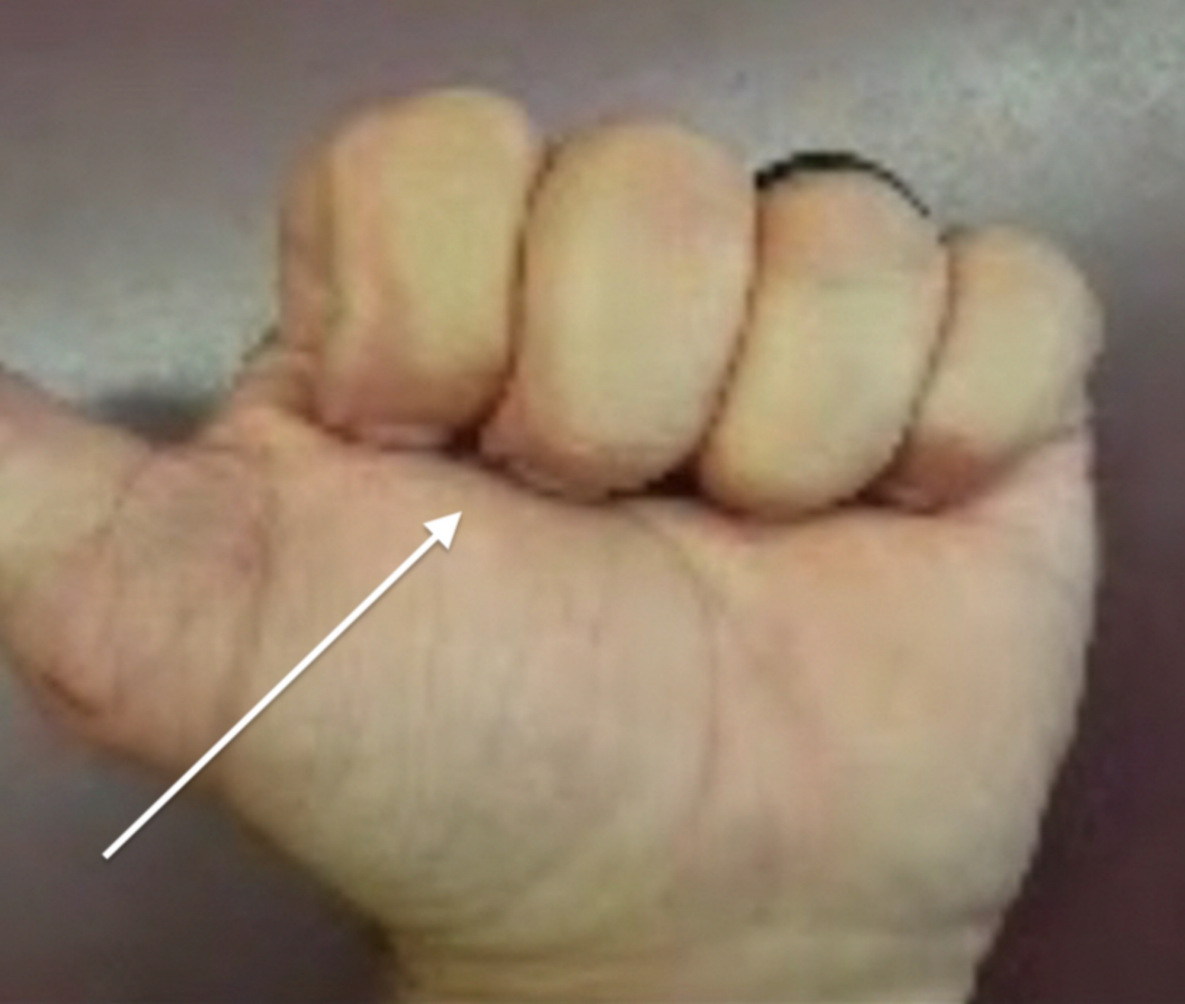
Full flexion without ring after three months

A 50-year-old male with no remarkable past medical history presented to the clinic for evaluation of a left middle finger injury sustained during jiu-jitsu practice two months prior. The patient’s finger was caught in a shirt sleeve during a takedown maneuver, and he subsequently felt and heard a “pop” in the palmar aspect of the finger. The patient reported pain with flexion and limited range of motion. A physical exam of the patient's hand demonstrated a reduced range of flexion for the middle finger and an inability to make a full composite fist with all fingers, with a 1-cm gap between the fingertip and hand when flexed fully (Figure 1). There was no tenderness over the area of the A2 pulley, and all flexor tendons were structurally intact. Application of moderate pressure to the palmar aspect of the affected finger utilizing a silicone wedding band allowed the patient to flex fully (Figure 2). This “Wedding Band Test” confirmed the suspected A2 pulley avulsion that was later visualized on magnetic resonance imaging (MRI) (Figure 3). The patient was then advised to continue wearing the silicone ring over the area of the A2 pulley for the next 3-4 months while avoiding any activity that placed undue stress over the injured area. During a one-year follow up, the patient reported that the injury had resolved and the full range of motion returned after roughly three months of wearing the silicone ring (Figure 4). 

## Discussion

Flexion of the digits is achieved by the action of the flexor digitorum superficialis (FDS) and flexor digitorum profundus (FDP) muscles. FDP originates from the body of the ulna and interosseous membrane while FDS originates from the medial epicondyle of the humerus. The tendons of FDS and FDP extend into the hand and attach to the base of the distal phalanges and middle phalanges. The flexor pulley system of the hand consists of a series of membranous and ligamentous “sheaths” through which the tendons of FDP and FDS course [1]. The integrity of these structures is essential for the full range of the motion of the fingers. There are a total of five pulleys located across each digit (A1-A5). The A2 pulley is located distal to the metacarpophalangeal (MCP) joints of digits 2 through 5, and functions to “hug” the flexor tendons close to the proximal phalanx, allowing it to essentially function as a fulcrum. The A2 and A4 pulleys are of significant importance and have been demonstrated to be the primary force bearing elements of the flexor pulley system and are critical for preventing bowstringing [5-6]. During a pinch or grasp, the A2 pulley is one of the strongest pulleys and withstands the most force. Other pulleys such as the A1, A3, and A5 exert less force on the tendon for the duration of the flexion [1].

Schoffl et al. proposed a grading system for the diagnosis and management of suspected pulley ruptures with recommendations for management. The lowest grade injuries involving minor strains and partial ruptures of the A2 pulley were successfully treated with conservative management, including immobilization and functional therapy. Ultrasonography was recommended as a first-line diagnostic tool, followed by MRI if the results were inconclusive [7].

In this case, positioning the ring over the region of the avulsion allowed the ring to serve in place of the A2 pulley. This successfully restored full flexion and the ability to make a composite fist. This “Wedding Band Test” could eliminate the need for an expensive and time-consuming ultrasound or MRI to confirm the presence of a suspected A2 pulley rupture. The utility of this test is also compounded by the fact that clinical evaluation of pulley ruptures is limited due to pain and edema that can obscure the defining characteristics of pulley ruptures such as bowstringing [8].

Schneeberger et al. developed a device called a pulley protection splint (PPS) that reduced the tendon-phalanx distance in a fashion similar to the silicone ring used in this case. This device functions effectively as a treatment option but may be impractical for use in clinical diagnosis [9].

## Conclusions

There is potential in the use of silicone bands for the accurate diagnosis of isolated A2 pulley avulsions and further study may be warranted. This method could be as accurate as traditional imaging methodologies while being less costly and potentially alleviating unnecessary healthcare expenditures.
